# Ultrasound-guided percutaneous microwave ablation of gallbladder polyps: A case report

**DOI:** 10.1097/MD.0000000000036622

**Published:** 2023-12-22

**Authors:** Huajiao Zhao, Yanwei Chen, Zheng Zhang, Mengyuan Shang, Yun Cai, Jingwen Ge, Xin Min, Xincai Wu, Shuangshuang Zhao, Baoding Chen

**Affiliations:** a Department of Ultrasound Medicine, Affiliated Hospital of Jiangsu University, Zhenjiang City, China.

**Keywords:** contrast-enhanced ultrasound, gallbladder polyps, microwave ablation, ultrasound guidance

## Abstract

**Rationale:**

Gallbladder polyps are a general term for localized lesions in which the gallbladder wall protrudes into the gallbladder cavity, and benign lesions are common. Although current guidelines recommend cholecystectomy for gallbladder polyps ≥ 10 mm in size, the probability of finding cancer in postoperative pathological specimens is low. We should avoid unnecessary cholecystectomy and treat polyps with gallbladder preservation. Microwave ablation is safe and effective for the treatment of solid lesions, and can inactivates polyps while preserving gallbladder. Hence, we report a case of ultrasound-guided percutaneous microwave ablation of gallbladder polyps.

**Patient concerns:**

A 72-year-old female patient had previously diagnosed a gallbladder polyp, but it was not taken seriously. Recently, the patient had occasional right upper abdominal discomfort and a desire to preserve gallbladder.

**Diagnoses:**

Ultrasound showed a medium hyperechoic papillary protrusion in the gallbladder without echo behind, and the changed position did not move. Contrast-enhanced ultrasound (CEUS) showed no malignant signs. The diagnosis was a gallbladder polyp.

**Interventions:**

The bile is drained and the drainage tube is fixed under real-time ultrasound guidance, then the gallbladder cavity is flushed and filled. Saline was injected between the serous and mucosal layers of the gallbladder to form an “edema band” to protect the gallbladder wall. Then, ultrasound-guided biopsy of gallbladder polyps was performed and sent for histological examination. Finally, the microwave needle was inserted into the target area under real-time ultrasonic guidance, and ablation was performed for 3 minutes (20 W). Postoperative CEUS: No significant enhancement was observed in the lesion.

**Outcomes:**

Within 6 months of follow-up, the patient’s gallbladder systolic function was normal, and there was no discomfort and no recurrence. The lesion reduction rate reached 100% at 1 week after surgery.

**Lessons:**

Ultrasound guided percutaneous microwave ablation of gallbladder polyps not only preserves the gallbladder but also inactivates the polyps without affecting the systolic function of the gallbladder, which provides a new idea for the treatment of gallbladder polyps.

## 1. Introduction

Gallbladder polyps are local lesions with or without pedicles protruding into the gallbladder cavity, most of which are benign. The most common nonneoplastic polyps are cholesterol polyps, accounting for approximately 60% to 90% of all gallbladder polyps, and most are asymptomatic.^[1–4]^ Adenomas are true neoplastic polyps with definite potential to develop into cancer, accounting for 4% to 7% of all gallbladder polyps.^[1]^ But only 6% of gallbladder cancers may be caused by polypoid lesions of the gallbladder.^[3]^ It is currently generally accepted that gallbladder polyps ≥ 10 mm in size have a high risk of malignancy.^[1,2,5]^ Therefore, gallbladder polyps ≥ 10 mm in size are usually recommended for cholecystectomy in daily practice.^[4,5]^ However, the gallbladder can store and secrete bile, which is involved in a variety of metabolic regulation.^[6,7]^ Hence, the removal of the gallbladder may lead to metabolic disorders.

Contrast-enhanced ultrasound (CEUS) is effective in distinguishing neoplastic polyps from nonneoplastic polyps with a sensitivity of 87.10%.^[8]^ Cholesterol polyps are usually late enhancement, and the arterial stage is equal enhancement. Adenomatous polyps frequently show continuous uniform enhancement and the arterial stage showed excessive enhancement. Gallbladder carcinoma often presents with uneven hyperenhancement in the arterial phase, which is quickly flushed away.^[1,3,8,9]^ In recent years, microwave ablation has been widely used in solid tumors. It has the advantages of high security, small intrusion and repeatability.^[10–12]^ In the past, gallbladder polyps were not the preferred ablation treatment, and few doctors attempted to ablate gallbladder polyps. Herein, we report a case of ultrasound-guided percutaneous microwave ablation of gallbladder polyps in a 72-year-old woman.

### 1.1. Consent

This study was approved by the ethics committee of Jiangsu university (approval no:KY2023H0621-05). This study did not involve vulnerable/minor population. The patient signed an informed consent form agreeing to publish this case report and any associated images.

## 2. Case report

**Figure 1.**
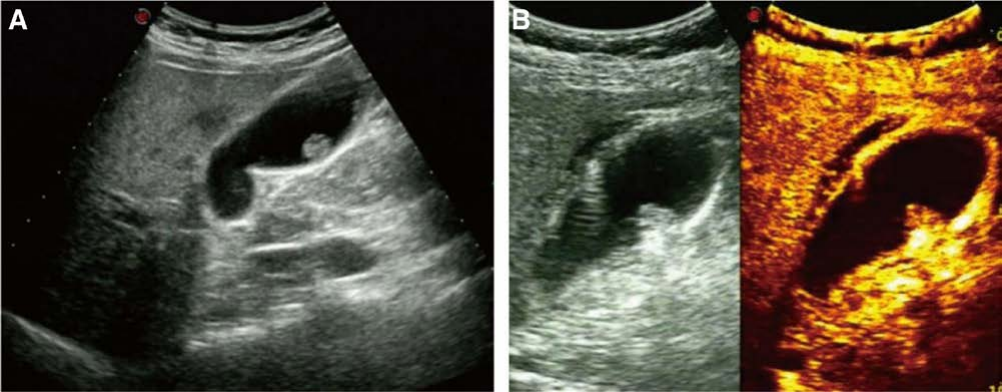
Preoperative ultrasound examination of the patient. (A) Conventional two-dimensional ultrasound showed a medium-high echo papillary eminence on the side wall of the gallbladder, with a wide base and a silent shadow in the back. It did not move when changing position. (B) Preoperative CEUS showed that the lesion was synchronously and uniformly high enhanced with the gallbladder wall in the arterial phase, and the enhancement speed was slightly faster than that of the surrounding liver parenchyma. The attached gallbladder wall was continuous and intact without thickening. CEUS = contrast-enhanced ultrasound.

A 72-year-old female was diagnosed with a gallbladder polyp on ultrasound several years prior, but no attention was given. Recently, the patient had occasional right upper abdominal discomfort. For further diagnosis and treatment, she was admitted to our hospital. After admission, ultrasound examination showed a medium hyperechoic papillary protrusion in the gallbladder, approximately 12 × 9 mm in size, with no echo behind, and the changed position did not move (Fig. 1A). Colour Doppler flow imaging (CDFI) showed that dotted blood flow signals could be detected inside the lesion. CEUS showed that the gallbladder polyp was enhanced synchronously and uniformly with the gallbladder wall in the arterial stage, and the enhancement rate was slightly faster than that of the surrounding liver parenchyma. The gallbladder wall at the attachment site had complete continuity and no thickening (Fig. 1B). Due to the patient’s strong desire to preserve the gallbladder, we intend to perform transcutaneous microwave ablation of gallbladder polyps under the guidance of ultrasound.

The preliminary laboratory results after admission were as follows: routine blood test: mean platelet volume of 13.2 fL, platelet distribution width of 19.4%, and the rest were not abnormal. Liver and kidney function: aspartate aminotransferase/alanine aminotransferase of 0.9, uric acid of 390 µmol/L, and the rest were not abnormal. Electrolytes, tumor markers and routine coagulation were all in the normal range.

**Figure 2.**
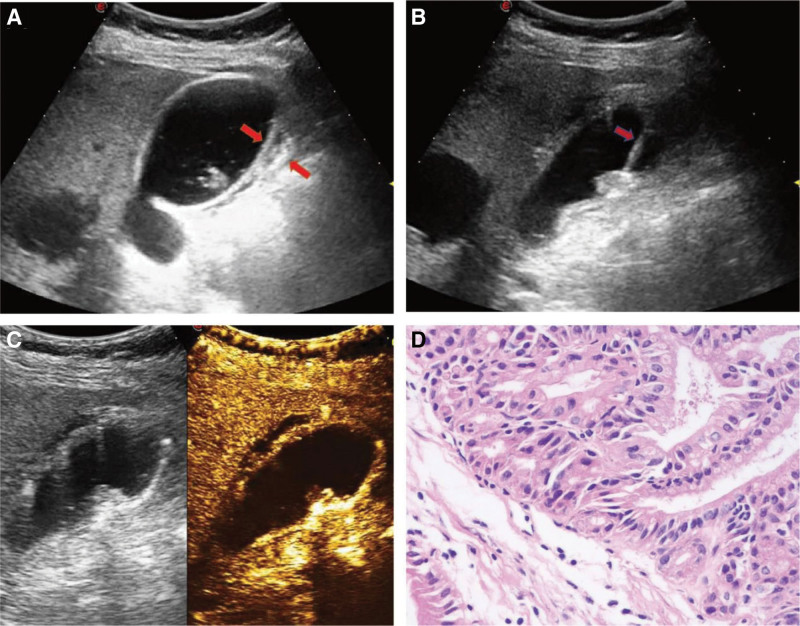
Ultrasound images of patients before and after ablation, as well as histopathologic image of ultrasound-guided biopsy of gallbladder polyps. (A) After the spacer fluid is injected into the serous layer and mucous layer of the gallbladder, the “edema band” is formed, which is between the 2 short red arrows. (B) Ultrasound-guided microwave ablation of gallbladder polyps (red arrow indicates microwave ablation needle). (C) Postoperative CEUS indicated no obvious enhancement of the lesion and good continuity of the gallbladder wall. (D) The histopathological findings suggest chronic cholecystitis with gallbladder adenoma (low grade). CEUS = contrast-enhanced ultrasound.

The operation was performed under general anesthesia. The puncture site was located on the right side of the upper abdomen. Under the guidance of real-time ultrasound, a pig caudal trocar (Bioteque Corporation, 6F, Taipei City, Taiwan Province) was punctured into the gallbladder, the needle core was extracted, yellow–green bile was extracted, the drainage tube was fixed, and was rinsed with normal saline several times. Then fill the gallbladder cavity with saline. Saline is injected between the serous and mucosal layers of the gallbladder to create edema (Fig. 2A). Ultrasound-guided biopsy of gallbladder polyps was performed, and a semiautomatic biopsy gun (HS Hospital Service S.p.A, 18G, Aprilia, Latina, Italia) was inserted from the base of the gallbladder to obtain a tissue sample. Under the guidance of real-time ultrasound, a 21G puncture needle was injected into the polyp from the base of the gallbladder, and lauromacrogol was injected. The microwave needle was inserted into the target area from the same path, and ablation was performed for 3 minutes (20 W) (Fig. 2B). Postoperative CEUS showed no significant enhancement was observed in the lesion and the continuity of the gallbladder wall was good (Fig. 2C). The operation was smooth, and no complications such as gallbladder perforation and bleeding occurred during or after the operation. Postoperative histopathology indicated that it was a gallbladder adenoma (Fig. 2D).

**Figure 3.**
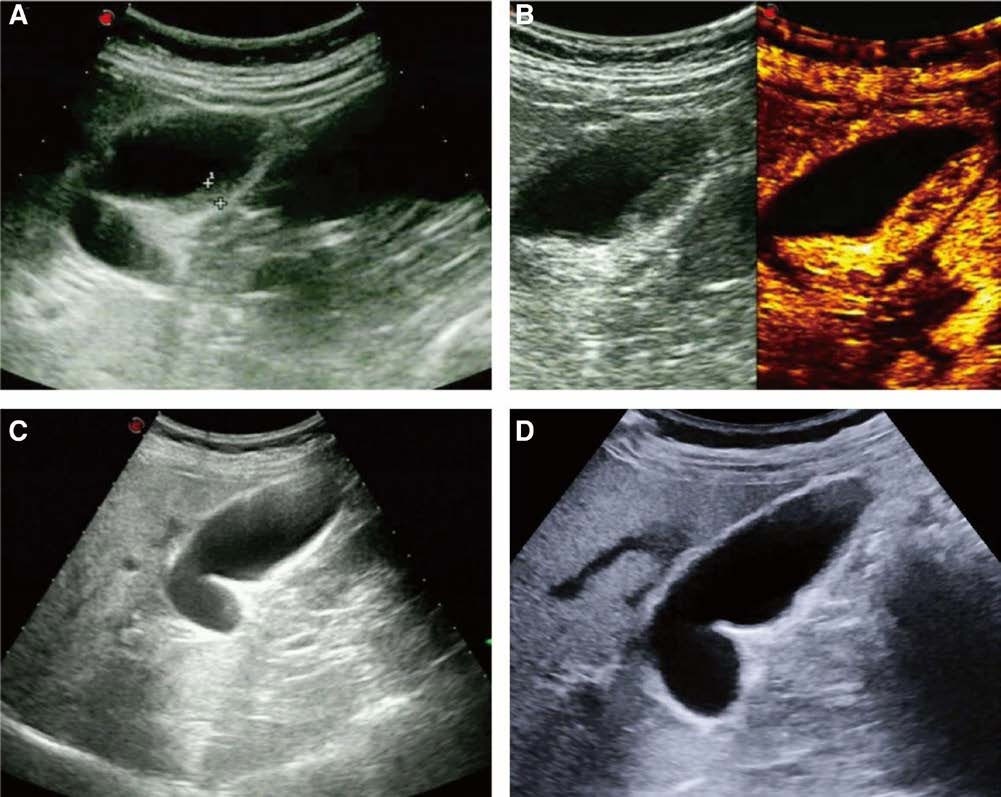
Postoperative follow-up ultrasound images of patients. (A) 1 week after surgery, the gallbladder wall was slightly rough, with local thickening up to 5.8 mm, and the internal echo was not uniform. (B) Postoperative 1 week, CEUS showed good continuity of the gallbladder wall and uneven high enhancement in the thickened area of the gallbladder wall. (C, D) 1 month and 3 months after surgery, ultrasound showed smooth gallbladder wall and no obvious abnormal echo in the cavity. CEUS = contrast-enhanced ultrasound.

One week after surgery, ultrasonography showed local thickening of the lateral wall of the gallbladder to 5.8 mm and uneven internal echo (Fig. 3A). CEUS indicated the continuity of the lateral wall of the gallbladder was good, and the thickening area of the lateral wall of the gallbladder showed uneven high enhancement (Fig. 3B). Ultrasonography at 1, 3, and 6 months after surgery all showed that the size of the gallbladder was within the normal range and the wall was smooth (Fig. 3C and D). The thickness of gallbladder wall was measured to be about 2 mm at 1 month after surgery and about 1 mm at 3 and 6 months after surgery (Table 1). The lesion disappeared 1 week after surgery, that is, the lesion shrinkage rate reached 100% (Table 1). Gallbladder emptying can be assessed with the fat meal test, reflecting gallbladder contractile function.^[13,14]^ The preoperative gallbladder shrinkage rate of the patient was 75%, and the postoperative gallbladder shrinkage rate was 78%, 72%, 74%, and 77% at 1 week, 1 month, 3 months, and 6 months, respectively (Table 1). The gallbladder contraction rate was > 50% before and after surgery, suggesting that ablation has no significant effect on gallbladder systolic function. During follow-up, no postoperative complications occurred and no recurrence of polyps (Table 1).

**Table 1:** Postoperative follow-up.

	Follow-up time (postoperative)			
	1 week	1 month	3 months	6 months
Postoperative complication (yes or no)	no	no	no	no
Lesion reduction rate (%)	100	100	100	100
Recurrent polyps (yes or no)	no	no	no	no
Gallbladder wall thickness (mm)	5.8	2	1	1
Gallbladder shrinkage rate (%)	78	72	74	77

Gallbladder shrinkage rate indicates gallbladder systolic function, ≥50% indicates normal gallbladder systolic function. Gallbladder shrinkage rate = (fasting gallbladder volume − post meal gallbladder volume)/fasting gallbladder volume × 100% and volume = 0.52 × L × W × H (where L is the long diameter of the longitudinal section of the gallbladder, W is the wide diameter of the transverse section, and H is the maximum diameter of the vertical wide diameter of the transverse section). lesion volume reduction rate = (lesion volume − volume at review)/lesion volume × 100% and the volume algorithm is the same.

## 3. Discussion

Most gallbladder polyps are benign cholesterol polyps, but a few neoplastic gallbladder polyps (NGPs) are at risk of malignancy, such as adenoma. Most guidelines recommend prophylactic cholecystectomy for symptomatic NGPs, NGPs ≥ 10 mm in diameter, or NGPs > 6 mm in diameter with other risk factors.^[4,15,16]^ Because the cancer rate of NGPs specimens is low, approximately 7% to 8.4%, not all NGPs should undergo cholecystectomy^[4]^ In recent years, microwave ablation has been widely used in solid tumors. It has the advantages of high safety, low invasiveness, short treatment time and so on.^[10,11]^ The application of the microwave ablation technique in the treatment of gallbladder polyps has the following advantages: ensuring the function of gallbladder; reducing the complications caused by excision and inactivating and removing polyps to reduce concerns.

Because the gallbladder is a hollow organ with thin walls, and thermal ablation may cause perforation. Once perforated, it can easily cause biliary peritonitis or septic shock.^[17]^ Therefore, we completely separated the serous layer and mucosal layer by injecting normal saline between them to form a “edema band” with a thickness greater than 10 mm. This “edema band” acts as a buffer, minimizing the occurrence of gallbladder perforation and increasing the safety of ablation. Meantime, the placement of a percutaneous transhepatic drainage tube is very important, and it is a safe and effective way to avoid complications caused by biliary leakage.^[18,19]^ During the operation, to prevent gallbladder perforation caused by heat injury and bile leakage, the bile should be drained in advance. After surgery, to prevent bile leakage due to high gallbladder tension or other reasons, it is still necessary to place a drainage tube to drain bile. The drainage tube is usually removed 1 week after surgery after the gallbladder wall has been determined to be free of damage.

Microwave ablation of gallbladder polyps can reduce the overtreatment of benign polyps, but may cause a delay in the treatment of malignant polyps. Studies have shown that the size of polyps is often overestimated pathologically, and surgical removal of polyps ≥ 10 mm leads to overtreatment of nonneoplastic polyps.^[3]^ For benign polyps, such as adenoma, microwave ablation therapy can avoid cholecystectomy. However, regular review should be conducted after surgery, and cholecystectomy should be performed immediately once malignant signs are found. Because for gallbladder cancer, surgical excision is the only treatment, and ablation alone can lead to disease progression.^[20]^ Therefore, it is critical to judge the benign and malignant of gallbladder polyps before operation.

At the same time, there are some limitations of this study. First of all, this single case lacks other cases as controls, which may have a certain occasionality. Therefore, large sample are still needed to further study the efficacy, prognosis and safety of ultrasound-guided percutaneous microwave ablation of gallbladder polyps. Second, the follow-up time for this patient was not long enough to determine whether the polyp would become other substances if it shed after ablation. We should lengthen the follow-up time of patients.

## 4. Conclusion

In conclusion, we successfully completed a case of ultrasound-guided percutaneous microwave ablation of gallbladder polyps, and the curative effect is good. Not only the gallbladder polyps were inactivated, but also the lesion shrinkage rate reached 100%. Meanwhile, microwave ablation did not affect the normal function of gallbladder after operation. The study provides a new idea for the treatment of gallbladder polyposis.

## Author contributions

**Data curation:** Yanwei Chen, Zheng Zhang.

**Investigation:** Mengyuan Shang, Yun Cai, Jingwen Ge, Xin Min, Xincai Wu, Shuangshuang Zhao.

**Methodology:** Huajiao Zhao, Baoding Chen.

**Project administration:** Yanwei Chen, Zheng Zhang, Baoding Chen.

**Supervision:** Baoding Chen.

**Writing – original draft:** Huajiao Zhao.

**Writing – review & editing:** Huajiao Zhao.
